# Weighting Strategies for Single-Step Genomic BLUP: An Iterative Approach for Accurate Calculation of GEBV and GWAS

**DOI:** 10.3389/fgene.2016.00151

**Published:** 2016-08-19

**Authors:** Xinyue Zhang, Daniela Lourenco, Ignacio Aguilar, Andres Legarra, Ignacy Misztal

**Affiliations:** ^1^Animal and Dairy Science, Animal Breeding and Genetics, University of GeorgiaAthens, GA, USA; ^2^National Agricultural Research InstituteLas Brujas, Uruguay; ^3^Institut National de la Recherche Agronomique, UMR1388 GenPhySECastanet-Tolosan, France

**Keywords:** genome-wide association, SNP window, WssGBLUP, BayesB, BayesC

## Abstract

Genomic Best Linear Unbiased Predictor (GBLUP) assumes equal variance for all single nucleotide polymorphisms (SNP). When traits are influenced by major SNP, Bayesian methods have the advantage of SNP selection. To overcome the limitation of GBLUP, unequal variance or weights for all SNP are applied in a method called weighted GBLUP (WGBLUP). If only a fraction of animals is genotyped, single-step WGBLUP (WssGBLUP) can be used. Default weights in WGBLUP or WssGBLUP are obtained iteratively based on single SNP effect squared (*u*^2^) and/or heterozygosity. When the weights are optimal, prediction accuracy, and ability to detect major SNP are maximized. The objective was to develop optimal weights for WGBLUP-based methods. We evaluated 5 new procedures that accounted for locus-specific or windows-specific variance to maximize accuracy of predicting genomic estimated breeding value (GEBV) and SNP effect. Simulated datasets consisted of phenotypes for 13,000 animals, including 1540 animals genotyped for 45,000 SNP. Scenarios with 5, 100, and 500 simulated quantitative trait loci (QTL) were considered. The 5 new procedures for SNP weighting were: (1) *u*^2^ plus a constant equal to the weight of the top SNP; (2) from a heavy-tailed distribution (similar to BayesA); (3) for every 20 SNP in a window along the whole genome, the largest effect (*u*^2^) among them; (4) the mean effect of every 20 SNP; and (5) the summation of every 20 SNP. Those methods were compared to the default WssGBLUP, GBLUP, BayesB, and BayesC. WssGBLUP methods were evaluated over 10 iterations. The accuracy of predicting GEBV was the correlation between true and estimated genomic breeding values for 300 genotyped animals from the last generation. The ability to detect the simulated QTL was also investigated. For most of the QTL scenarios, the accuracies obtained with all WssGBLUP procedures were higher compared to those from BayesB and BayesC, partly due to automatic inclusion of parent average in the former. Manhattan plots had higher resolution with 5 and 100 QTL. Using a common weight for a window of 20 SNP that sums or averages the SNP variance enhances accuracy of predicting GEBV and provides accurate estimation of marker effects.

## Introduction

Genomic best linear unbiased prediction (GBLUP) and Bayesian methods are routinely used for genomic selection (GS) and genome-wide association studies (GWAS) in animal and plant breeding, and in human disease studies. The main objective of GWAS is to identify the quantitative trait loci (QTL) with major effect, whereas GS focuses on using genomic information to better evaluate the genetic potential of individuals. Genomic estimated breeding value and single nucleotide polymorphism (SNP) effect or variance can be easily derived from both GBLUP and Bayesian methods. GBLUP assumes a normal distribution for SNP effects, and calculates their effect from phenotypes and a genomic relationship matrix. When not all individuals in a population are genotyped, single-step GBLUP (ssGBLUP) is the method of choice (Aguilar et al., 2010). According to Legarra et al. (2014), the extra information on non-genotyped animals, the ability to account for pre-selection, and the independency of pseudo-phenotypes are partially responsible for gains in accuracy over other genomic methods. Bayesian methods such as BayesA, BayesB, and BayesC (Meuwissen et al., 2001; Kizilkaya et al., 2010; Habier et al., 2011) are non-linear approaches that usually assign to SNP effect a heavy tailed prior distribution, and then sample from the posterior distribution via Markov chain Monte Carlo (MCMC). GBLUP and ssGBLUP usually assumes equal weights for all SNP (Meuwissen et al., 2001; VanRaden, 2008; Goddard and Hayes, 2009). This assumption is biologically incorrect but makes the statistics robust by limiting the number of unknown parameters (Meuwissen et al., 2001). Nonlinear methods such as BayesA and BayesB assume heterogeneous variances of SNP effects, with emphasis on the SNP that links to major effects (Meuwissen et al., 2001; Meuwissen and Mike, 2004). The performance of these methods has been proven to be better than GBLUP approaches in simulation studies assuming a few QTL with large effects and many QTL with small or null effects (Meuwissen et al., 2001; Meuwissen and Mike, 2004; Lund et al., 2009; Guo et al., 2010). However, experiences with real dairy cattle data indicate that Bayesian regressions have resulted in reduced accuracy because of ignoring SNP with small effects (Cole et al., 2009; Su et al., 2010) and that GBLUP approaches performed well for the majority of the traits of interest in livestock, mainly because of their polygenic nature (Hayes et al., 2009; VanRaden et al., 2009; Aguilar et al., 2010; Chen et al., 2011; Forni et al., 2011; Wang et al., 2014).

One way to account for locus-specific variance in GBLUP-based methods is to include different weights for SNP. If those weights are known, weighted GBLUP (WGBLUP) provides genomic estimated breeding value (GEBV) similar to those of a Bayesian procedure using the same weights (Legarra et al., 2009). WGBLUP and WssGBLUP were developed to allow the estimation of weights within GBLUP or single-step GBLUP (ssGBLUP), respectively. Many studies have found advantages of WGBLUP-based methods compared to unweighted GBLUP (Snelling et al., 2011; Gao et al., 2012; Tiezzi and Maltecca, 2014). Sun et al. (2011) proposed two iterative procedures for calculating weights in WGBLUP, where the iteration was used to mimic the MCMC sampling in which both the prior and the posterior distributions kept being updated. In the first procedure, the weights were calculated as $w_{j}^{\left(i+1\right)}={û}_{j}^{\left(i\right)2}$, where $w_{j}^{\left(i+1\right)}$ is the weight of SNP *j* at iteration *i*+1 and ${û}_{j}^{\left(i\right)}$ is the effect of SNP *j* at iteration *i*. This procedure is effective for identifying top QTL but excessively shrinks small SNP effects; thus, the accuracy of GEBV is reduced. In the second procedure the weights were calculated as $w_{j}^{\left(i+1\right)}={û}_{j}^{\left(i\right)2}+t$, where $t=\;\frac{\sigma_{g}^{2}}{2\sum_{j=1}^{m}p_{j}q_{j}},\sigma_{g}^{2}$ is the genetic variance; *p* and *q* are the major and minor allele frequencies at locus *j*, respectively, and *m* is the number of SNP. This procedure introduced a constant to avoid SNP with no effect and brought the accuracy of GEBV close to that by BayesC but yielded Manhattan plots with lower resolution.

Recently, it was found that assigning a common weight to markers on a chromosomal region yielded more accurate estimates of GEBV under GBLUP-based methods (Wang et al., 2012; Su et al., 2014). Su et al. (2014) used group-marker variance from BayesR as a weighting factor for GBLUP in the evaluation of a dairy cattle population. Reliabilities for 4 production traits and mastitis were, on average, up to 1% higher and bias was reduced by 11% when using the mean variance of 30-SNP window compared with single SNP weighting. Xu (2013) demonstrated improved predictability in diploid plant QTL mapping using an artificial bin of linkage disequilibrium (LD)-linked neighboring markers.

Wang et al. (2012) evaluated WssGBLUP with simulation data using $d_{i\left(t+1\right)}=u_{i\left(t\right)}^{2}\left[2p_{i}\left(1-p_{i}\right)\right]$, where *d*_*i*(*t*+1)_ is the weight of SNP *i* at iteration *t*+*1*, $u_{i\left(t\right)}^{2}$ is the effect of SNP *i* at iteration *t*, and *p*_*i*_ is the minor allele frequency (MAF). They iterated either on SNP alone or on GEBV and SNP. The first procedure gave a good identification of top QTL, and the second procedure provided a slightly higher accuracy of GEBV compared to BayesB, but only at the second iteration. The same study also found that the correlation of simulated and estimated SNP effects was lower than the correlation between simulated SNP and a sum of the effect of 8 SNP clustered around simulated QTL.

We hypothesized that efficient SNP weighting methods could considerably increase accuracy of predicting GEBV and help to better estimate SNP effects under WssGBLUP models. Therefore, the objective of this study was to present new procedures to calculate SNP weights individually or for genomic regions (windows) that would improve genomic predictions at animal and SNP level in WssGBLUP compared to other genomic models.

## Materials and methods

### Data simulation

To test our hypothesis, one trait with a mean of 1.0, a phenotypic variance of 2.0, and an heritability of 0.5 was simulated using QMSim (Sargolzaei and Schenkel, 2009). All the genetic variance was caused by QTL. A total of 20 chromosomes were created with an average length of 82 cM and containing 45,000 evenly distributed SNP with MAF ≥ 0.05. Three scenarios were considered involving different numbers of randomly placed QTL (5, 100, and 500) to mimic simple traits defined by major effects and complex traits affected by numerous minor effects. The scenarios were named 5-QTL, 100-QTL, and 500-QTL. All QTL were selected among SNP, and their effects were sampled from a gamma distribution with a shape parameter of 0.4. Both SNP and QTL were bi-allelic with no overlap between their positions, and a mutation rate of 2.5 × 10^−5^ was assumed for markers and QTL per generation per locus. The simulated population originated from a historical population with 1000 generations of random mating to create linkage disequilibrium (LD) and establish mutation-drift balance. After that, a recent population was established from 200 males and 2600 females out of the historical population. The simulation was carried out for 205 generations. In every generation, the above number of males and females were selected to mate and produce 1 offspring, forming a population with an effective size (Ne) of 743. For all the analyses, generations 200–204 were treated as a training population and generation 205 as a validation population, with 1240 and 300 genotyped animals respectively. The complete dataset contained 18,400 individuals in the pedigree, of which 13,000 were phenotyped and 1540 were genotyped. Phenotypes were the sum of general mean, true breeding values (sum of QTL effects) and random residuals. Each simulated scenario was replicated 10 times.

### WssGBLUP and new weighting methods

The ssGBLUP uses SNP to construct the genomic relationships. The genomic relationship matrix in our study was created as in VanRaden (2008):

$$G=\;\frac{\text{ZDZ}\prime }{2\sum p_{i}(1-p_{i})},$$

where *p*_*i*_ is the MAF of SNP *i*, **Z** is a matrix of centered genotypes, and **D** is a diagonal matrix of weights, where *d*_*ii*_ is the weight for SNP *i*. In regular GBLUP-based methods, **D** = **I**, which gives a weight of 1 to all SNP. Strandén and Garrick (2009) derived conversions of GEBV into SNP effects under GBLUP models and Wang et al. (2012) extended the approach to ssGBLUP. In this approach, weight for SNP is based on SNP effect, and the latter is a best linear unbiased prediction derived from GEBV:

$$\hat{u}=\;DZ^{\prime }G^{-1}\hat{g},$$

where $\hat{u}$ is a vector of estimated SNP effects and $\hat{g}$ is a vector of GEBV. The weight for SNP *i* can be calculated as $u_{i}^{2}$ or $u_{i}^{2}2p_{i}\left(1-p_{i}\right)$, and in the current study we used the first way.

Improvements in the SNP weights can be obtained iteratively either by recomputing only the SNP effects or by recomputing SNP effects and GEBV (Wang et al., 2012). The latter also improves predictions of GEBV and was chosen for this study. The default WssGBLUP calculates and uses SNP-specific weights. Following Wang et al. (2012), the iterative steps in WssGBLUP were:

- Set *t* = 1, **D**^(*t*)^ = **I** and $G^{\left(t\right)}=\;\frac{{\text{ZD}}^{\left(\text{t}\right)}\text{Z}\prime }{2\sum p_{i}(1-p_{i})}$
- Compute ${\hat{g}}^{\left(t\right)}$ using the ssGBLUP approach
- Compute SNP effects as ${\hat{u}}_{i}{}^{\left(t\right)}=\;D^{\left(t\right)}Z^{\prime }{\left(G^{\left(t\right)}\right)}^{-1}{\hat{g}}^{\left(t\right)}$
- Calculate SNP weight as ${d_{ii}}^{\left(t+1\right)}=\;{û}_{i}^{2}$, for all SNP *i*
- Normalize **D**^(*t*+1)^
- $G^{\left(t+1\right)}=\frac{ZD^{\left(t+1\right)}Z^{\prime }}{2\sum p_{i}(1-p_{i})}$
- *t* = *t* +1
- Iterate from b) until *t* −1 = 10

Whereas the approach derived by Wang et al. (2012) calculates SNP effects based on GEBV which combines direct genomic value (DGV; or sum of SNP effects weighted by SNP content), parent average (PA), yield deviation (YD), progeny contribution (PC), and pedigree prediction (PP). Lourenco et al. (2015a) showed that DGV is a more appropriate starting point for calculating SNP effects, because genotyped populations may comprise animals with different levels of accuracy. Therefore, in the step (c) GEBV was replaced by DGV.

For all the 5 new procedures, the changes were done in step (d) where SNP weights are calculated based on SNP effects. Therefore, instead of using $d_{ii\;}=\;{û}_{i}^{2}$ as SNP weight, the 5 new procedures we propose are:

1. Constant: still using the concept of SNP-specific weights, the weight was calculated as
  $$\begin{array}{l} d1_{ii}=\;{û}_{i}^{2}+c \end{array}$$
  where $c=\text{max}\left({û}_{i\left(0\right)}^{2}\right)$ is a constant chosen as the top SNP weight in the first iteration;
2. Nonlinear A: using similar approach to VanRaden (2008), SNP-specific weights were calculated as
  $$\begin{array}{l} d2_{ii}=\;{û}_{i}^{2}/v^{\left|s_{i}-2\right|} \end{array}$$
  where ν is a scale parameter standing for the departure from normality and *s*_*i*_ is the number of standard deviations from the mean for each $2\;\sum^{}p_{i}\left(1-p_{i}\right)$;
3. Largest window: uses the concept of SNP-window weights, with weights for a group of 20 SNP as
  $$\begin{array}{l} d3_{i,i}=d3_{i+1,i+1}=\ldots =d3_{i+19,i+19}=\text{max}\left(u_{i}^{2},\ldots ,\;u_{i+19}^{2}\right) \end{array}$$
  where *d*3_*ii*_ with *ii* from i to i+19 was used as weight for all SNP in the window comprised of 20 SNP; it uses non-overlapping windows;
4. Mean window: the weight for a group of 20 SNP was calculated as
  $$d4_{ii}=d4_{i+1,i+1}=\ldots =d4_{i+19,i+19}{=}^{\sum_{i=1}^{n}u_{i}^{2}}/n$$
  where *n* is the size of the non-overlapping window;
5. Summed window: the weight for a group of 20 SNP was calculated as
  $$d5_{ii}=d5_{i+1,i+1}=\ldots =d5_{i+19,i+19}=\;\sum_{i=1}^{n}u_{i}^{2}$$

### Models and computation

Quality control of genomic data retained SNP with call rates >0.9, minor allele frequencies >0.05, and departures from Hardy-Weinberg equilibrium (difference between expected and observed frequency of heterozygous) <0.15. An average of 36,000 SNP remained after quality control, and the average LD (r2 = correlation between loci pair) in the last generation was about 0.29.

For WssGBLUP, the model included population mean, animal effect, and random residual error. All phenotypes (except for validation animals), pedigree, and genotypes were accounted for. GEBV was obtained by using the software BLUPF90 (Misztal et al., 2002) with the simulated variance components; SNP effect was obtained by postGSf90 (Aguilar et al., 2010; Wang et al., 2012). A total of 10 iterations of BLUPF90 combined with postGSf90 were used for the default WssGBLUP and WssGBLUP with new weighting methods. Each run of postGSf90 updated weights for SNP, whereas each run of BLUPF90 used the updated weights to constructed G matrices and, consequently, to improve GEBV estimates.

For GBLUP, the model was exactly as the one used in WssGBLUP, however, only genotypes and phenotypes for genotyped animals (except validation animals) were used together with the simulated variance components. The software BLUPF90 (Misztal et al., 2002) was used assuming the animals were unrelated through the pedigree.

The model for BayesB and BayesC had similar effects to the model used for WssGBLUP, but the animal effect was replaced by SNP effects. The phenotypic information was estimated breeding value (EBV) de-regressed (EBV_DP_) following the approach described in Garrick et al. (2009), assuming that 0.05 of the genetic variation was not explained by the markers. The marker effects were assumed to follow a normal distribution with *u*_*i*_ ~ N$\left(0,\sigma_{u_{i}}^{2}\right)$, and $\sigma_{u_{i}}^{2}$ the variance of the *i*^th^ SNP. The proportion of SNP having no effect was set to 50%, 90% or 99%. Priors for $\sigma_{u_{i}}^{2}$ were calculated as $\frac{\sigma_{a}^{2}}{2\sum^{}p_{i}\left(1-p_{i}\right)}$, where $\sigma_{a}^{2}$ is the total simulated genetic variance. Degrees of freedom for the SNP and residual variances were set to 4 and 10, respectively. The software GenSel (Fernando and Garrick, 2009) was used to calculate SNP effects under the BayesB and BayesC approaches. A Monte Carlo Markov Chain (MCMC) was run for 41,000–50,000 and 1000–10,000 iterations were discarded as burn-in. For BayesB, 10 Metropolis-Hasting iterations were run per MCMC. Estimates of SNP effects were based on the posterior means of the MCMC.

For WssGBLUP, the output for genotyped animals in the validation population was GEBV that contained DGV, PA, PP; whereas for GBLUP, GEBV contained only DGV. The output for BayesB and BayesC is in the format of SNP effect, which can be translated into DGV by multiplying a row vector of SNP content by a column vector of SNP effects. In this way, GEBV from Bayesian methods for validation animals contained only DGV.

### Model comparison

The 5 new weighting procedures for WssGBLUP were compared with GBLUP, BayesB and BayesC with the proportion of markers with no effect (π) set to 0.5, 0.9, and 0.99. The simulated values were used as benchmark. Two comparisons were made: (1) accuracy of predicting GEBV defined as the correlation between true simulated breeding value (TBV) and GEBV in the validation population (“accuracy” hereinafter); (2) ability to detect simulated QTL, done by visual inspection of Manhattan plots and by the amount of genetic variance explained. We did not derive significance thresholds or *P*-value for the latter comparison method because the objective was to identify the ability of the new weighting procedures in tracking the position simulated QTL, disregarding the significance of their effect on the trait. In addition, several studies reported difficulty in obtaining a measure of SNP significance when using shrinkage methods or Bayesian methods (Servin and Stephens, 2007; Wakefield, 2007, 2009).

## Results and discussion

### Estimation of GEBV

Tables 1–3 show accuracies of GEBV and standard errors for the 5 new procedures to calculate SNP weights in WssGBLUP under three QTL scenarios, along with default WssGLBUP. Table 4 shows the accuracies of GEBV for GBLUP, BayesB, and BayesC. The average accuracies of the 5 new procedures were 0.87, 0.80, and 0.77 under 5−, 100−, and 500-QTL scenarios, respectively, and the standard deviation among 10 iterations ranged from 0.02 to 0.07. With default weighting, the accuracy increased initially but declined after some iterations depending on the number of simulated QTLs. As the number of QTLs increased, the inflection point came earlier (on iterations 4, 3, and 2 for 5−, 100−, and 500-QTL scenarios, respectively). The decline in accuracy with iteration was the result of continuously adding weight to SNP with large effects while shrinking SNP with small effects. Consequently, the accuracy of GEBV gradually decreased with iteration because the number of SNP with small effect increased.

**Table 1:** **Average accuracy of genomic estimated breeding values (GEBV) and standard deviation across 10 replicates per iteration, for the 5-QTL scenario under the default weighting and 5 new weighting procedures for single-step Genomic Best Linear Unbiased Prediction (ssGBLUP)**.

**5-QTL**	**Iterations**									
**Procedure**	**1**	**2**	**3**	**4**	**5**	**6**	**7**	**8**	**9**	**10**
Default	0.80	0.86	0.90	**0.91**	0.91	0.90	0.90	0.90	0.90	0.90
	0.04	0.02	0.03	0.04	0.04	0.05	0.05	0.05	0.05	0.05
Constant	0.80	0.83	0.86	**0.88**	0.88	0.88	0.88	0.88	0.88	0.88
	0.04	0.03	0.03	0.03	0.03	0.03	0.03	0.03	0.03	0.03
Nonlinear A	0.80	0.80	0.80	0.81	0.81	0.82	0.82	0.82	0.82	**0.83**
	0.04	0.04	0.03	0.03	0.02	0.02	0.03	0.03	0.03	0.03
Largest window	0.80	0.85	**0.91**	0.91	0.90	0.90	0.90	0.90	0.89	0.89
	0.04	0.02	0.03	0.04	0.05	0.05	0.05	0.05	0.05	0.05
Mean window	0.80	0.85	0.91	**0.92**	0.91	0.92	0.92	0.92	0.92	0.92
	0.04	0.02	0.03	0.04	0.05	0.04	0.04	0.04	0.04	0.04
Sum window	0.80	0.85	0.90	**0.91**	0.91	0.91	0.91	0.91	0.91	0.91
	0.04	0.02	0.03	0.05	0.05	0.05	0.05	0.04	0.04	0.04

The greatest accuracy at the earliest iteration is in bold face.

**Table 2:** **Average accuracy of genomic estimated breeding values (GEBV) and standard deviation across 10 replicates per iteration, for the 100-QTL scenario under the default weighting and 5 new weighting procedures for single-step Genomic Best Linear Unbiased Prediction (ssGBLUP)**.

**100-QTL**	**Iterations**									
**Procedure**	**1**	**2**	**3**	**4**	**5**	**6**	**7**	**8**	**9**	**10**
Default	0.79	0.82	**0.83**	0.81	0.81	0.80	0.80	0.80	0.80	0.79
	0.02	0.02	0.02	0.03	0.03	0.03	0.03	0.03	0.03	0.03
Constant	0.79	0.81	**0.83**	0.83	0.83	0.83	0.83	0.83	0.83	0.83
	0.02	0.02	0.02	0.02	0.02	0.02	0.02	0.02	0.02	0.02
Nonlinear A	0.79	0.79	**0.80**	0.80	0.80	0.80	0.80	0.80	0.80	0.80
	0.02	0.02	0.02	0.02	0.02	0.02	0.02	0.02	0.02	0.02
Largest window	0.79	0.81	**0.83**	0.82	0.79	0.77	0.76	0.76	0.75	0.75
	0.02	0.02	0.02	0.02	0.03	0.03	0.03	0.03	0.03	0.03
Mean window	0.79	0.81	**0.84**	0.83	0.82	0.81	0.81	0.80	0.80	0.80
	0.02	0.02	0.02	0.02	0.02	0.03	0.03	0.03	0.03	0.03
Sum window	0.79	0.82	**0.84**	0.82	0.80	0.79	0.79	0.78	0.77	0.77
	0.02	0.02	0.02	0.02	0.03	0.03	0.04	0.04	0.04	0.04

The greatest accuracy at the earliest iteration is in bold face.

**Table 3:** **Average accuracy of genomic estimated breeding values (GEBV) and standard deviation across 10 replicates per iteration, for the 500-QTL scenario under the default weighting and 5 new weighting procedures for single-step Genomic Best Linear Unbiased Prediction (ssGBLUP)**.

**500-QTL**	**Iterations**									
**Procedure**	**1**	**2**	**3**	**4**	**5**	**6**	**7**	**8**	**9**	**10**
Default	**0.81**	0.81	0.79	0.77	0.76	0.76	0.75	0.75	0.75	0.75
	0.04	0.04	0.04	0.05	0.05	0.05	0.06	0.06	0.06	0.06
Constant	**0.81**	0.81	0.81	0.81	0.81	0.81	0.81	0.81	0.81	0.81
	0.04	0.04	0.04	0.04	0.04	0.04	0.04	0.04	0.04	0.04
Nonlinear A	**0.81**	0.81	0.81	0.81	0.81	0.81	0.81	0.81	0.81	0.81
	0.04	0.04	0.04	0.04	0.04	0.04	0.04	0.04	0.04	0.04
Largest window	**0.81**	0.81	0.81	0.78	0.74	0.71	0.69	0.67	0.66	0.65
	0.04	0.04	0.04	0.04	0.05	0.05	0.06	0.06	0.06	0.06
Mean window	**0.81**	0.81	0.81	0.78	0.75	0.73	0.71	0.70	0.69	0.69
	0.04	0.04	0.04	0.05	0.05	0.06	0.06	0.06	0.06	0.07
Sum window	**0.81**	0.81	0.81	0.78	0.74	0.72	0.70	0.69	0.68	0.67
	0.04	0.04	0.04	0.05	0.05	0.05	0.05	0.05	0.05	0.06

The greatest accuracy at the earliest iteration is in bold face.

**Table 4:** **Average accuracy of genomic estimated breeding values (GEBV) for BayesB and BayesC under 3 simulation scenarios with varying π**.

**Method**	**π^a^**	**5-QTL**		**100-QTL**		**500-QTL**	
		**Accuracy**	**SE**	**Accuracy**	**SE**	**Accuracy**	**SE**
GBLUP	0.00	0.77	0.08	0.77	0.07	0.67	0.10
BayesC	0.50	0.66	0.05	0.61	0.04	0.63	0.10
	0.90	0.75	0.06	0.67	0.03	0.65	0.09
	0.99	0.87	0.04	0.76	0.07	0.68	0.07
BayesB	0.50	0.88	0.07	0.65	0.06	0.42	0.17
	0.90	0.89	0.07	0.68	0.07	0.41	0.16
	0.99	0.90	0.07	0.72	0.07	0.47	0.15

a π is the proportion of SNP that have no effect.

For early iterations (≤5), largest, mean, and summed windows were the most accurate methods. Using the mean weight of 20 SNP in the windows as weight for the entire window improved accuracy by 0.01–0.09 compared to the other new procedures under the 5-QTL scenario. For 100-QTL, the procedure that added a constant outperformed the other new procedures, whereas for 500-QTL both constant and nonlinear A achieved the greatest accuracies that persisted for 10 generations. Overall, we observed that window procedures performed better than procedures with single SNP weights because the uncertainty was smaller (Su et al., 2014) and it also avoided extremely small values for SNP weights. A window size of 20 SNP was chosen over 5, 10, 50, and 100 based on accuracy (results not shown). Many factors, including the size of the reference population and population structure, influence the optimum window size (Su et al., 2014). Window procedures maintained high accuracy with 5 QTL but lost the superior performance in late iterations with more QTL. The largest window scenario decreased in accuracy fastest among all window procedures, especially under the 500-QTL scenario, because it gave the greatest weight to the windows with large SNP effects and least weight to those with small SNP effects. This over- and under-weighting introduced bias into the solutions. In regard to real genetic evaluations of massive data, the performance of iterations higher than 3 may not matter because one iteration usually takes from several hours (simple models) up to several days (complex model and population structure).

One of the purposes of investigating new weighting methods was to avoid sudden drops in accuracy after the peak was reached. We observed that most of the new methods were able to fulfill this requirement, especially when the number of simulated QTL was small. When the number of QTL increased, the method that adds a constant to û^2^ was the only one able to hold prediction accuracies over iterations. The main reason is that the constant value was chosen as the greatest û^2^ in the first iteration and remained the same for all iterations. This indicates that the shrinkage occurring along iterations is an important cause for the drop in reliability when SNP weights are calculated iteratively. The best constants for 5-, 100-, and 500-QTL scenarios were 8, 40, and 13, respectively, as they showed the highest accuracy averaged among 10 iterations compared to other tested constants. These values avoided SNP with no effects, which could reduce the accuracy of GEBV, while not deviating large effects significantly. Although adding a constant did not give as high an accuracy at early iterations as the window procedures, the accuracy remained stable after the peak was reached at iteration 5 (for 5- and 100-QTL). The accuracy for the default procedure was exceeded by the constant only in the 100-QTL scenario (+0.01). Adding a constant to avoid under-weighting in the 5-QTL scenario, where most SNP did not have effect on the trait, was redundant and counterproductive. Still, in the procedure that added a constant, the plateaued accuracies exceeded GBLUP by 0.11, 0.06, and 0.14 under the 5-, 100-, and 500-QTL scenarios (Tables 1–4). On average, the increase was greater than the 0.06 reported in Sun et al. (2011) that used WGBLUP in a similar simulation of 10,000 SNP and 33 QTLs. The drawback of this approach is that the mechanism behind picking the right constant is still empirical and unclear; e.g., the average genetic variance *t* derived from GBLUP in Sun et al. (2011) was possibly too small for ssGBLUP. Theoretically, a threshold between zero and the peak SNP effect increases the bottom line of the absolute value for SNP with no effects. This threshold should both guarantee high accuracy of EBV and differentiate SNP effects. Number and distribution of QTL effects are related to this threshold, but in reality these are unknown.

The nonlinear A weighting method used in our study was adapted from VanRaden (2008) who defined the weight of SNP *i* as $1.25^{\left|s_{i}-2\right|},$ where *s*_*i*_ is the number of standard deviations from the mean, and 1.25 represents the departure from normality. In our study, *s* ranged from 1.06 to 1.12. This procedure gave more weight to SNP with smaller effects, thus preventing the drastic decrease in accuracy. Its inferior performance compared with other procedures occurred for two reasons. First, oligogenic traits are correlated with few large QTL and the mean effect of all QTL is close to zero. Secondly, nonlinear A assigns more weight to SNP with effects but not to those with no effects; thus, it introduces bias into GEBV. This study showed that nonlinear A only performed as well as some of the other procedures (maximum accuracy of 0.81) under the 500-QTL scenario, whereas for 5-QTL scenario it had the lowest accuracy among WssGBLUP approaches.

The accuracies of GEBV from WssGBLUP were compared with those from Bayesian methods (Tables 1–4). Except for the 5-QTL scenario, all WssGBLUP procedures under all scenarios surpassed BayesC and BayesB in accuracy before iteration 7. Accuracies for BayesB and BayesC π = 0.99 were the same under the 5-QTL scenario. For this scenario, BayesB with 3 different values of π and BayesC π = 0.99 outperformed WssGBLUP with nonlinear A weights. BayesC with π = 0.99 was 7 percentage points lower compared with the peak accuracy of the default procedure under the 100-QTL scenario (0.76 vs. 0.83) and 13 percentage points lower under the 500-QTL scenario (0.68 vs. 0.81). The biggest difference between default WssGBLUP and BayesB was under the 500-QTL scenario when π was 0.99 (0.81 vs. 0.41). This is consistent with previous studies (Daetwyler et al., 2010; van den Berg et al., 2015), which indicated that Bayesian methods perform well when the number of QTL is small, whereas GBLUP-based methods perform better when the number of QTs is large, because it assumes an infinitesimal model. Single step GBLUP also includes pedigree relationships that contribute to the accuracy of GEBV (Legarra et al., 2009; Aguilar et al., 2010; Christensen and Lund, 2010), whereas DGV from Bayesian methods exclude parent average (Garrick et al., 2009). The accuracy of GBLUP (Table 4) that had both pedigree and parent average removed was most of the times outperformed by BayesB or BayesC. Sun et al. (2011) also found that accuracies for BayesB and BayesC were 2.5 and 9.9% higher compared with GBLUP. In our study, posterior variances from BayesB and BayesC were used to weight **G** in WssGBLUP, but it did not improve the accuracy of GEBV (results not shown). When SNP variance from the literature was used as SNP weight for GBLUP, Zhang et al. (2014) showed considerable increase in accuracy for most of the analyzed traits in dairy cattle.

Lourenco et al. (2015b) showed that GEBV from ssGBLUP is composed of parent average, phenotypic information, contribution from progeny, DGV, and pedigree prediction (pedigree relationships among genotyped animals). The accuracy of GEBV depends, therefore, on the weights each of these components receive. On the other hand, the accuracy from BayesB, BayesC, and GBLUP depends only on DGV, but if the same variance is used for all SNP in GBLUP, weights for DGV will not be optimized.

When methods that use SNP window weighting (largest window, mean window, and summed window) were compared to single SNP weighting (constant and nonlinear A), we observed an average increase in accuracy of 5 percentage points for 5-QTL (Tables 1–4). For 100- and 500-QTL, there was a decrease in accuracy when window weighting was applied (0.80 vs. 0.81 and 0.74 vs. 0.81, respectively). When looking at the third iteration, which was the inflection point for most of the SNP window methods, the difference between windows and single SNP increased to 8 percentage points in 5-QTL. Hassani et al. (2015) observed that a SNP window of 1 SNP had the worst performance in terms of accuracy, and an increased SNP window helped to better capture the QTL signal when few QTL are simulated. In our 5-QTL scenario, largest and mean window of 20 SNP had better performance than the default weighting method, especially after the second iteration, although the difference was small (1% point). Mean window 20 was also able to maintain accuracy along the 10 iterations.

Differences between the first iteration (**D** = **I**) and the iteration where the peak accuracy was reached reduced as the number of simulated QTL increased, confirming that weights are more important when traits are influenced by fewer QTL. Thus, for the majority of the traits of interest in livestock breeding, ssGBLUP is able to attain the greatest accuracies without using weights for SNP. For specific traits influenced by few QTL or traits known for being influenced by important QTL (e.g., DGAT1 in milk fat and milk protein in dairy cattle) using weights that are common to a SNP window may help to better capture the signal from the QTL in that region. Therefore, using weights for SNP windows helps to improve the accuracy when a relatively small number of markers lose the ability to capture the possible QTL effects. Possibly, differential SNP weighting better depicts the covariance structure of **G** and reflects the actual contribution of SNP to additive genetic variance of a trait (Su et al., 2014).

### QTL identification

**Figure 1:**
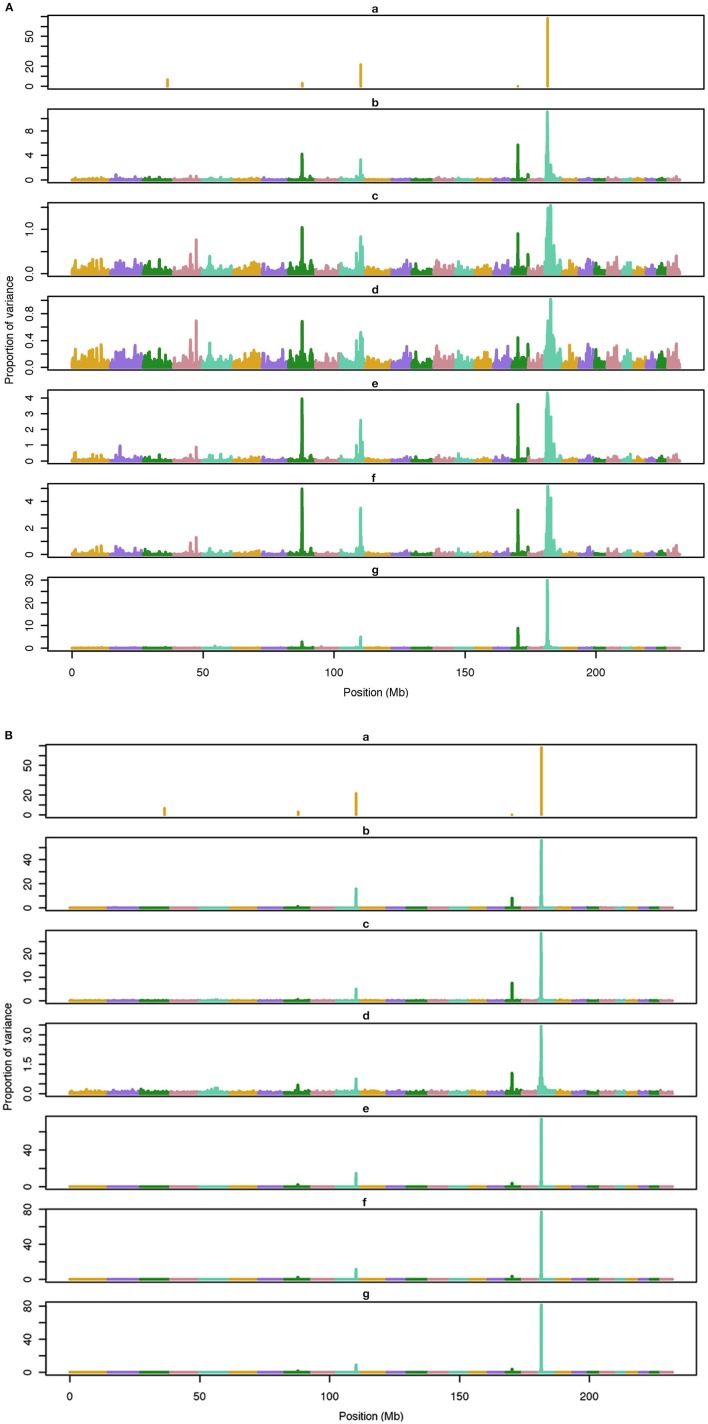
**Proportion of variance (%) explained by simulated QTL and SNP for different methods under 5-QTL simulation**. **(A) (a)**, true QTL; **(b)**, default; **(c)**, constant; **(d)**, nonlinear A: weights as ν^|*s*−2|^, where ν is a scale standing for the departure from normality, and s is number of standard deviation from mean for each $u_{i}^{2}$; **(e)**, largest window; **(f)**, mean window; **(g)**, sum window. **(B) (a)**, true QTL; **(b)**, BayesC π = 0.5; **(c)**, BayesC π = 0.9; **(d)**, BayesC π = 0.99; **(e)**, BayesB π = 0.5; **(f)**, BayesB π = 0.9; **(g)**, BayesB π = 0.99.

**Figure 2:**
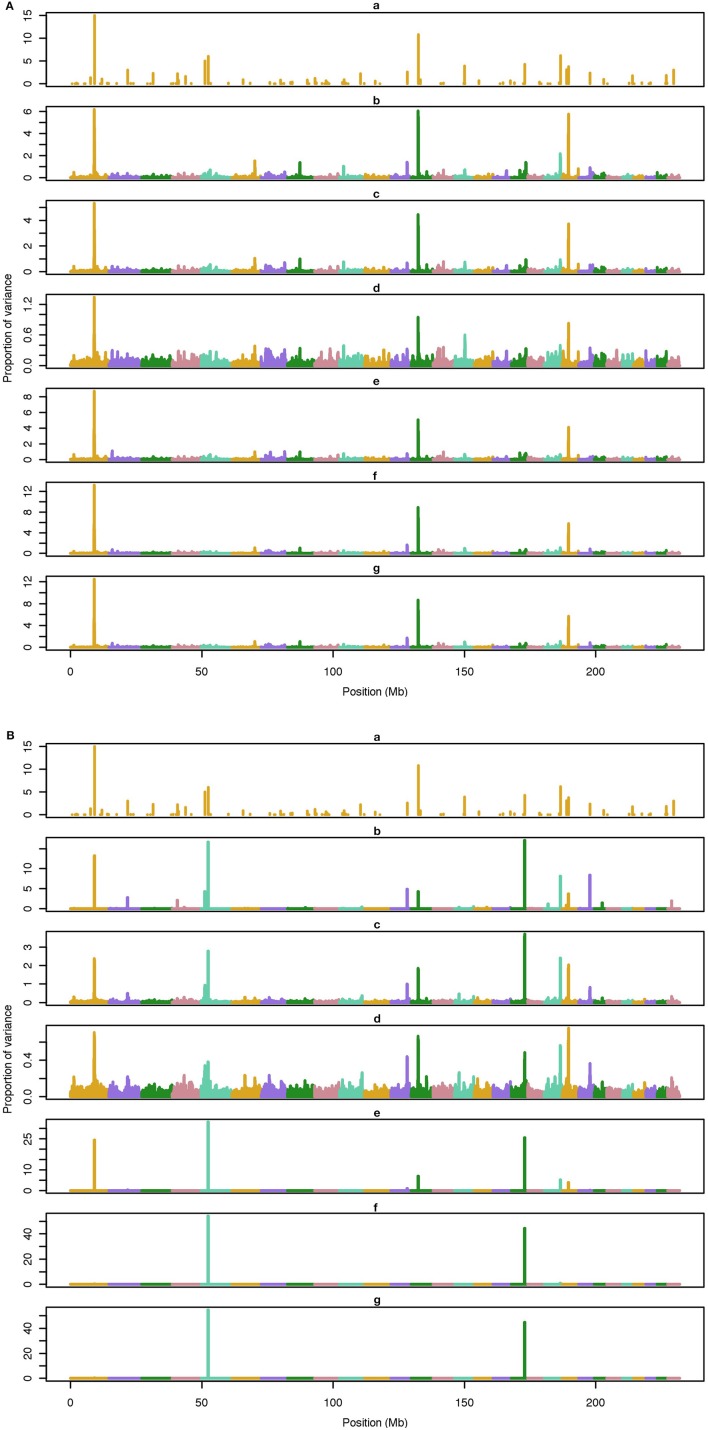
**Proportion of variance (%) explained by simulated QTL and SNP for different methods under 500-QTL simulation**. **(A) (a)**, true QTL; **(b)**, default; **(c)**, constant; **(d)**, nonlinear A: weights as ν^|*s*−2|^, where ν is a scale standing for the departure from normality, and s0 is number of standard deviation from mean for each $u_{i}^{2}$; **(e)**, largest window; **(f)**, mean window; **(g)**, sum window. **(B) (a)**, true QTL; **(b)**, BayesC π = 0.5; **(c)**, BayesC π = 0.9; **(d)**, BayesC π = 0.99; **(e)**, BayesB π = 0.5; **(f)**, BayesB π = 0.9; **(g)**, BayesB π = 0.99.

**Figure 3:**
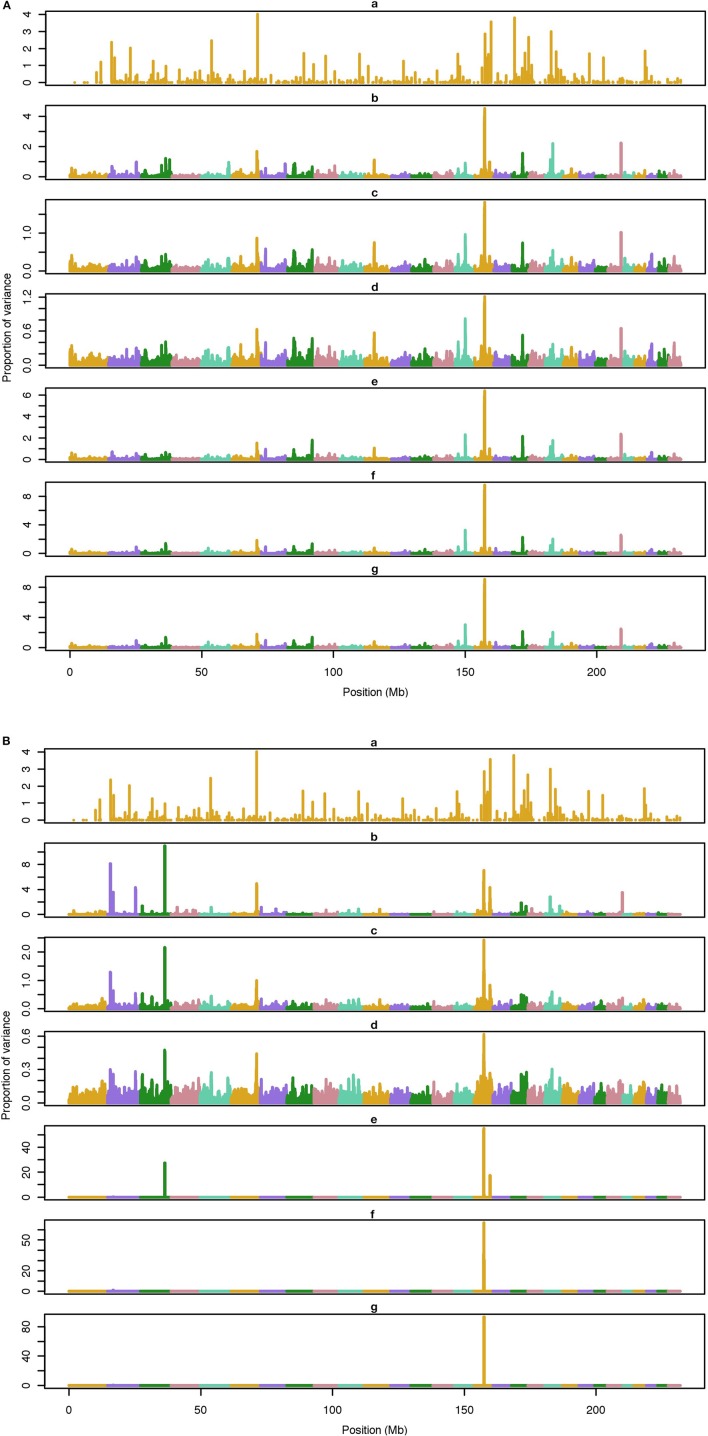
**Proportion of variance (%) explained by simulated QTL and SNP for different methods under 100-QTL simulation**. **(A) (a)**, true QTL; **(b)**, default; **(c)**, constant; **(d)**, nonlinear A: weights as ν^|*s*−2|^, where ν is a scale standing for the departure from normality, and s is number of standard deviation from mean for each $u_{i}^{2}$; **(e)**, largest window; **(f)**, mean window; **(g)**, sum window. **(B) (a)**, true QTL; **(b)**, BayesC π = 0.5; **(c)**, BayesC π = 0.9; **(d)**, BayesC π = 0.99; **(e)**, BayesB π = 0.5; **(f)**, BayesB π = 0.9; **(g)**, BayesB π = 0.99.

The average number of QTL with major effect that account for 50% of the total variance in 5-, 100-, and 500-QTL scenarios were 1.5 (0.5), 6.0 (2.0), and 26.1 (2.8). Figures 1–3 show Manhattan plots for SNP effects in 5-, 100,- and 500-QTL. Figures 1A, 2A, 3A show effects for default and proposed weights for WssGBLUP, whereas Figures 1B, 2B, 3B show effects for default WssGBLUP, BayesB, and BayesC. Under all QTL scenarios, using weights for SNP window produced clearer Manhattan plots than the single SNP weights. Although up to 20% of the simulated QTL were not correctly detected, most QTL with large effects were identified and few peaks were spurious associations. The good performance of window procedures resulted from assigning equal weights to neighboring SNP. This is especially instrumental to scenarios with many effects that are likely to be correlated with the majority of the QTL. The procedure with a constant reduced the difference between large and small SNP effects; hence, the plot was of lower resolution and had many small peaks compared to the default WssGBLUP. This was expected because SNP with small effect had a boost due to the extra weight added. A similar pattern was found in the nonlinear A procedure because the weighting factors had a limited range for all SNP. We observed that the shrinkage of SNP effects was bigger for the constant and nonlinear A methods under all scenarios. In the 500-QTL scenario, single SNP weighting caused an underestimation of SNP effects and SNP window weighting caused an overestimation, whereas default WssGBLUP returned SNP effects of the same scale as the simulated QTL. These results confirm that using weights for SNP when the trait is influenced by several QTL is not beneficial for better estimating SNP effects. If the trait is influenced by few QTL, using the maximum, average or sum of effects in a window of 20 SNP would help to capture clear signals from QTL. Bayesian methods, especially BayesB, best detected the simulated QTL under the 5-QTL scenario, however, with a different scaling of the SNP effect. The scaling of the SNP effect was very variable under the 5-QTL scenario. Under the 100- and 500-QTL scenarios, BayesB captured <1% of the SNP and assigned extremely high variance to them, especially in the 500-QTL scenario. This is because of the extreme shrinkage and avoidance of SNP with small effects, which caused bias in estimating SNP variances (Calus and Veerkamp, 2007). Furthermore, BayesB and BayesC patterns depended on the choice of π.

Applying common weights for groups of SNP can help to increase the resolution of Manhattan plots; however, the resolution may be limited by the effective number of independent markers as proposed by Li et al. (2012). In their human genetics study, they showed that, when the ratio of effective number of markers to total number of markers is high (i.e., less redundant SNP), the threshold to declare a SNP as significant is more stringent, which indicates that the resolution worsens. Pocrnic et al. (2016) showed that the number of independent markers relates to the effective population size (*Ne*), where small populations share less independent markers. Perhaps, a Manhattan plot with high resolution (with noisy pattern) is a feature of smaller genotyped populations.

## Conclusion

New procedures for calculating SNP weights in WssGBLUP can be effective in improving both the accuracy of GEBV and SNP effects. GEBV from WssGBLUP are more accurate than those from BayesB and BayesC, although different priors and π for the latter can change the ranking of the methods. Procedures that consider weights for a SNP window may be the best choice compared to single SNP given that the true number of QTL may not be known in real data. In addition, considering a group of SNP in the same genomic region may be the most appropriate way to capture the signal of an unknown QTL. The WssGBLUP method is especially useful for GWAS and GEBV estimation when only a fraction of the population is genotyped. WssGBLUP with 3 iterations may be enough to reach maximum predictivity of GEBV and SNP effects when a trait is influenced by few QTL.

## Author contributions

XZ implemented the research, contributed ideas to the algorithms, and wrote the article. DL contributed ideas to the algorithms and revised the article. IA programmed the original version of software, interpreted the algorithms, and revised the article. AL contributed ideas to the algorithms and revised the article. IM lead and designed the project, contributed ideas to the algorithms, and revised the article. All authors approved the final version to be published, and agreed to be accountable for all aspects of the work in ensuring that questions related to the accuracy or integrity of any part of the work are appropriately investigated and resolved.

## Funding

USDA's National Institute of Food and Agriculture (Washington, DC; Agriculture and Food Research Initiative competitive grant 2015-67015-22936).

### Conflict of interest statement

The authors declare that the research was conducted in the absence of any commercial or financial relationships that could be construed as a potential conflict of interest.
